# Online education for prosthetics and orthotics students in the era of COVID-19 pandemic in Iran: challenges, opportunities, and recommendations

**DOI:** 10.1186/s12909-023-04339-5

**Published:** 2023-05-16

**Authors:** Maryam Jalali, Vahideh Moradi, Taher Babaee, Gholamreza Aminian, Parviz Mojgani, Saeed Shahabi

**Affiliations:** 1 Center for Educational Research in Medical Sciences (CERM), Department of Medical Education, School of Medicine, Iran University of Medical Sciences, Tehran, Iran; 2 Rehabilitation Research Center, Department of Orthotics and Prosthetics, School of Rehabilitation Sciences, Iran University of Medical Sciences, Tehran, Iran; 3 Iran-Helal Institute of Applied Sciences and Technology, Tehran, Iran; 4 Research Center for Emergency and Disaster Resilience, Red Crescent Society of the Islamic Republic of Iran, Tehran, Iran; 5 Department of Orthotics and Prosthetics, University of Social Welfare and Rehabilitation Sciences, Tehran, Iran; 6 Health Policy Research Center, Institute of Health, Shiraz University of Medical Sciences, Shiraz, Iran

**Keywords:** Online education, Online learning, Prosthetics and orthotics, Rehabilitation, Iran

## Abstract

**Introduction:**

With the advent of the COVID-19 pandemic, many higher education programs in Iran, including prosthetics and orthotics (P&O), had to shift to the online environment all at once. This unanticipated transition was challenging for the educational system. However, online education is superior in some aspects to conventional methods, and this situation may offer opportunities. This study was carried out from September 2021 to March 2022 to investigate the challenges and opportunities of online education in the P&O sector in Iran based on the opinions of students and faculty members. Relevant recommendations will also be discussed.

**Methods:**

In this qualitative study, semi-structured interviews were conducted in both oral and written formats. Purposive and snowball sampling techniques were used to recruit undergraduate and postgraduate P&O students, as well as P&O faculty members, for this qualitative study. The data gathered from interviews with study participants were analyzed by thematic analysis.

**Results:**

Based on the data analysis, many sub-themes of the three main themes were recognized: (1) challenges: technical, socioeconomic, environmental distractors, supervision and evaluation, workload, digital competence, interactions, motivation, sessions-related issues, class time, hands-on and clinical training; (2) opportunities: technological innovations, infrastructure development, flexible learning environment, student-centered learning, availability of contents, time and cost saving, high concentration, more self-confidence; (3) recommendations: technical infrastructure, team dynamics, hybrid courses, time management, awareness.

**Conclusion:**

Online education of P&O during the era of the COVID-19 pandemic was accompanied by a series of challenges. Technical issues and the gravity of hands-on training in this field were significant challenges. This era, however, provided the opportunity to facilitate the establishment of needed infrastructure and support technological innovations for online education. Considering hybrid (mixed online and on-site) courses was recommended to improve the quality of learning.

## Introduction

Following the COVID-19 pandemic, social distancing was carried out in most parts of the world in an effort to reduce the spread of the virus [1]. Many public activities, including those of universities, were limited or suspended [2–4]. There was no exception to this rule in medical universities and faculties of rehabilitation sciences, given the high risk of transmission of the infection in related settings [5, 6]. In this situation, many universities moved to online education within a short time [7]. The sudden and unplanned transition from conventional methods to online education has been fraught with challenges that might have negative impacts on the quality of education [8]. On the other hand, some experts argue that the trend of education, even before the COVID-19 pandemic, both in the clinical fields and in the theoretical part, has been towards the use of new technologies and remote teaching [9], and COVID-19 has had a promoting effect in a short period [10].

Education in the field of prosthetics and orthotics (P&O), as one of the rehabilitation disciplines, encompasses two sectors of theoretical and practical-clinical courses [11]. Furthermore, as in other medical sciences, the internship is unquestionably an essential component of P&O training [11]. In Iran, the history of the field of P&O dates back to 1967, when the Iranian Red Crescent Society set up training courses and then established a specialized clinic in Tehran [12]. For the first time, in 1984, the Faculty of Rehabilitation Sciences and Social Welfare, which is currently one of the faculties of Iran University of Medical Sciences (IUMS), accepted students for the associate degree. Then in 1987, this course became a bachelor’s degree [11, 12]. After many years and numerous revisions, the field of P&O is currently taught in six universities and at three levels of bachelor’s, master’s, and Ph.D. in Iran [12].

Before the COVID-19 pandemic, none of the P&O departments had a background in using online education, and in particular, they lacked experience in distance teaching for practical courses. With the advent of the pandemic, universities sought to provide online education platforms for their departments. These platforms, in general, possessed the same basic features for all departments and, although helpful in theoretical training, did not enjoy the needed capacity for practical courses. Hands-on training as a key component of education in the field of P&O could not be covered by the usual platforms, and special arrangements should have been made to carry on practical courses. To continue the education process, however, these measures, with all their advantages and disadvantages, were experienced during the COVID-19 pandemic.

Studying various aspects of online education is not a recent subject [13], and this issue has attracted the attention of researchers since the early 1990s. With the pronounced shift to online education in response to the COVID-19 pandemic, there was an excellent opportunity to study this topic more readily. Researchers explored the integration of online teaching and learning in different health professions. This study aims to investigate the challenges and opportunities of the online education of P&O during the COVID-19 pandemic in Iran based on the opinions of students and faculty members. In addition, recommendations provided by students and faculty members to improve the experience of online education will be discussed. Looking into this experience and the lessons learned from this situation can be helpful for future planning.

## Methods

### Study design

We conducted this qualitative study using a phenomenological approach from September 2021 to March 2022 in Iran. Indeed, this research design provides a favorable opportunity to gain a deeper understanding of participants’ views on a particular phenomenon. Under- and post-graduate P&O students and P&O faculty members were recruited to attend in semi-structured interviews. The research team considered both Consolidated criteria for reporting qualitative research (COREQ) items [14] and Standards for reporting qualitative research (SRQR) criteria [15] throughout the study to promote the reporting quality.

### Study setting

This study was done by a group of researchers, mainly from three departments of P&O in Iran. The Research Ethics Committee of the IUMS provided the ethical approval for this study (IR.IUMS.REC.1400.494). Informed consent for participating in this study was obtained from all the participants before the interview sessions.

### Sampling and recruitment strategy

The recruiting process was initiated with purposive sampling, and then snowball sampling was applied to select other participants. To cover the greatest diversity, the research team attempted to recruit individuals from different universities. Our inclusion criteria were under- and post-graduate P&O students and P&O faculty members who participated in online learning during the COVID-19 pandemic. After initial contact with the potential sample, a formal invitation, which included an informed consent form and general information about the objectives of the research project as well as the research team, was sent to the individual via email or WhatsApp. In addition, study information was given orally to the participant at the beginning of each interview. The informed consent form also stated that the participant could leave the study at any stage with full authority. The recruiting process continued until data saturation was reached. Three interviews with duplicate data were considered to ensure that data saturation was achieved and new information would no longer be obtained.

### Data collection

Semi-structured interviews were done in oral and written formats by two authors (TB and VM). First, face-to-face interviews were conducted in a quiet environment with the presence of only the interviewer and the interviewee. Then, in cases where face-to-face interviews were not possible, questions were sent to the participants in audio files, and they were asked to submit their answers orally or in writing. To ease the interview process, an interview guide containing a number of open-ended questions was used during the interviews (Table 1). Interview questions were reviewed and revised based on the feedback received from the initial interviews for greater transparency. In addition to digitally recording interview sessions, the interviewer took notes. At the end of each interview, the recorded files were transcribed verbatim and saved in Office Word software.

**Table 1:** Interview guide

Open-ended questions
1. What challenges did you face while learning online during COVID-19 pandemic?
2. What are the opportunities and benefits of online education during COVID-19 pandemic?
3. What are your recommendations for improving online education?
4. What is your proposed style? In-person education or online education or combined mode?

### Data analysis

To analyze the collected data, Colaizzi’s phenomenological approach was used [16]. Based on the Colaizzi approach, first, two researchers (SSH and MJ) read the text frequently for more familiarity. Then, the authors began coding the meaning units manually. In the next step, relationships among achieved codes were examined, and each was placed in a related category. In the end, the categories were reviewed and merged to form the main themes of the study. The data analysis process was performed in parallel with data collection. In the event of any disagreement at this stage, the discussion and the participation of the third author (PM) were used.

### Rigor and trustworthiness

A number of strategies have been introduced to promote the rigor and trustworthiness of qualitative studies [17]. In accordance with Guba and Lincoln’s recommendation [18], five criteria, including credibility, dependability, confirmability, transferability, and authenticity, can be considered throughout the qualitative studies. In this study, the research team adopted several approaches to meet these criteria: (1) evaluating the results by relevant experts (credibility); (2) participating several authors with different scientific backgrounds in data analysis (dependability); (3) checking the results by involving samples (confirmability); (4) considering highest diversity in recruiting samples (transferability); and (5) inserting citations from almost all participants (authenticity) [18].

## Results

Out of 31 invited individuals, three students and one faculty member refused to participate in the interviews due to their busy schedule. In final, 27 individuals including 23 P&O students and four P&O faculty members participated in this qualitative study. The characteristics of the participants have been shown in Table 2. Following the data analysis process, the findings were categorized in accordance with the three main themes: challenges, opportunities, and recommendations (Table 3). The findings will be described in detail below, along with direct quotes from participants.

**Table 2:** Characteristics of participants

ID	Gender	Age (years)	Education status	University	Residence	Interview format
S01	Female	20	BCs	IRCIHE	-	Oral
S02	Male	22	BCs	IRCIHE	-	Oral
S03	Female	24	MSc	IUMS	-	Oral
S04	Female	20	BCs	IRCIHE	-	Oral
S05	Male	21	BCs	IRCIHE	-	Oral
S06	Female	22	BCs	IUMS	-	Text
S07	Male	19	BCs	IRCIHE	-	Oral
S08	Female	20	BCs	IRCIHE	-	Oral
S09	Female	19	BCs	IRCIHE	-	Text
S010	Male	19	BCs	IRCIHE	-	Text
S011	Female	20	BCs	IRCIHE	-	Oral
S012	Female	20	BCs	HUMS	-	Text
S013	Female	No Information	PhD	IUMS	-	Text
S014	Female	19	BCs	IRCIHE	-	Voice
S015	Male	23	MSc	USWRS	Tehran	Text
S016	Male	30	PhD	EUMS	Tehran	Text
S017	Male	31	PhD	EUMS	Esfahan	Text
S018	Female	26	MSc	IUMS	Tehran	Text
S018	Female	24	MSc	USWRS	Tehran	Text
S019	Male	31	PhD	EUMS	Esfahan	Text
S020	Female	25	MSc	IUMS	Rasht	Text
S021	Male	19	BSc	IUMS	Ardabil	Oral
S022	Male	20	BSc	IUMS	Lorestan	Oral
S023	Male	24	BSc	IUMS	Rasht	Oral
F01	Male	No Information	Assistant professor	-	Tehran	Text
F02	Female	No Information	Assistant professor	-	Tehran	Text
F03	Female	No Information	Assistant professor	-	-	Text
F04	Male	No Information	Assistant professor	-	Tehran	Text

*Abbreviations:* *IRCIHE* Iranian Red Crescent Institute of Higher Education, *IUMS* Iran University of Medical Sciences, *HUMS* Hamedan University of Medical Sciences, *USWRS* University of Social Welfare and Rehabilitation Sciences, *EUMS* Esfahan University of Medical Sciences, *KUMS* Kerman University of Medical Sciences

**Table 3:** Summary of themes and sub-themes

Themes	Sub-themes	Codes
Challenges	Technical	“…the low capacity of the Internet in our country [Iran] has caused our online meetings to be associated with serious disruptions.”[S05] “Sometimes the power goes out. This prevents us from accessing the class…”[S09]
	Socioeconomic	“The price of a laptop has gone up a lot. I know many of my friends who cannot afford to buy an appropriate laptop!” [S019]
	Environmental distractors	“The family does not notice at all that I have an online class. I’m at the class being called…” [S010]
	Supervision and evaluation	“Online tests are not really fair … the response time is very short” [S022] “I do not think online exams are appropriate because some students cheat and we cannot accurately assess their performance.” [F01]
	Workload	“Creating slides and videos is really time consuming. The bulk of my time is devoted to preparing educational content.” [F02]
	Digital competence	“Interestingly, many professors are unfamiliar with how to use online platforms. Sometimes it takes several minutes to take a screen sharing!” [S06]
	Interactions	“…, interactions between students and faculty members are not well established.” [S016]
	Motivation	“There are so many one-sided online meetings that bore us. I think the professor himself/herself gets tired of this style of teaching!” [S012]
	Sessions-related issues	“Often meetings do not start at a pre-arranged time. This causes our plans to be disrupted and mental conflicts to arise for us!” [S023]
	Hands-on and clinical training	“In the future, advances in technology may help to hold clinical classes virtually, but at present, existing technologies cannot replace student interaction with patients in clinical environment.” [F04]
Opportunities	Technological innovations	“The online courses have led to the use of new methods to convey educational content, such as developing applications that use artificial intelligence to simulate body movements.” [S017]
	Infrastructure development	“Despite the shortcomings that still exist, online classes have led to the provision of new electronic devices and tools that have increased the quality of education.” [F02]
	Flexible learning environment	“Online education makes it possible for us to attend classes with any physical condition. It is very appropriate from this perspective.” [S03]
	Student-centered learning	“Online education has led us to visit more educational and research sites and use the educational materials provided in them. Somehow, it has increased students’ self-learning” [S015]
	Availability of contents	“Even if we are absent from the class, we still have easy access to the content of the class …” [S08]
	Time and cost saving	“We no longer have to be in so much traffic to get to university. Now we come to class very easily from home… It saves us both time and money!” [S020]
	High concentration	“My concentration during online meetings is very good. On the other hand, online meetings make me ask questions of the teacher with higher confidence.” [S01]
	More self-confident	
Recommendations	Technical infrastructure	“In my opinion, new systems and software should be created to facilitate clinical education in this period.” [F01]
	Team dynamics	“Students need to work more closely together in online sessions and help resolve their own issues through member-checking.” [F03]
	Hybrid courses	“It is really hard to learn clinical topics online. I think it is better to have most clinical sessions in-person.” [S07]
	Time management	“Both students and faculty members need to be more committed to starting online classes on time.” [S011]
	Awareness	“More training and orientation classes should be held to familiarize faculty and students with how to use online platforms.” [S019]

### Theme 1: challenges

The participants raised numerous technical issues making their online sessions difficult. The very slow speed of the Internet was one of the challenges that many students, as well as faculty members, criticized. In fact, the unfavorable speed of the Internet causes online meetings to face serious disruptions. One of the students stated:

“Unfortunately, the increasing use of the Internet during the COVID-19 pandemic and the low capacity of the Internet in our country [Iran] has caused our online meetings to be associated with serious disruptions.” [S05]

Furthermore, power outages were another issue pointed out during the interviews, which made access to online meetings a serious problem. In this regard, one of the participants explained:

“Sometimes the power goes out. This prevents us from accessing the class….” [S09]

Notably, some faculty members stated that they did not have the up-to-date tools to prepare and present content online. So, this has made it difficult for them to have an attractive online presentation.

“Although there is always an emphasis on making online classes attractive, we do not have many tools and programs!” [F03]

In addition, the poor performance of e-learning platforms was discussed by many participants. Participants believed that platforms designed to provide online training had very poor capacity and service.

“Unfortunately, these platforms where online classes are held have very few features and their capacity been very low. Interestingly, they sometimes crash.” [S014]

Socioeconomic issues were other common challenges raised in this qualitative study. The dramatic increase in the price of electronic equipment such as laptops, smartphones and even Internet tariffs has caused some participants to face serious problems purchasing them. In this regard, one student said:

“The price of a laptop has gone up a lot. I know many friends who cannot afford an appropriate laptop!” [S019]

During the interview sessions, participants repeatedly mentioned the negative effects of environmental distractors on online learning. For instance, one student pointed out the undesirable family environment and believed that family members did not care about their online classes.

“The family does not care that I have an online class. I’m at the class being called…” [S010]

Furthermore, some faculty members criticized the inappropriate environment of teaching online. The lack of proper sound insulation and privacy in the professors’ rooms made them feel uncomfortable.

“The professors’ rooms in our college are very close to each other and any conversation can be heard. This prevents concentration and distracts us ….” [S019]

One of the challenges mentioned by many participants, both students and faculty members, was the lack of proper supervision and assessment through online education. On the one hand, the students believed that the assessments done through online tests were unfair, while the professors believed that such assessments were not very real because of the possibility of fraud.

“Online tests are not really fair ... the response time is very short” [S022]

“I do not think online exams are appropriate because some students cheat, and we cannot accurately assess their performance.” [F01]

Holding classes online has led teachers to spend more time preparing educational materials. As a result, the workload of professors has increased significantly. Some professors explained this issue during the interview sessions.

“Creating slides and videos is really time-consuming. The bulk of my time is devoted to preparing educational content.” [F02]

In addition, a number of faculty members described that their lack of familiarity with digital tools has made it difficult for them to work appropriately. Some students also pointed out this problem and stated that some faculty members are not very familiar with the tools used in online education.

“Interestingly, many professors are unfamiliar with how to use online platforms. Sometimes it takes several minutes to take a screen sharing!” [S06]

Online education has greatly influenced both interactions among students and interactions between students and faculty members. This issue was mentioned in most of the interview sessions.

“Because humans are social beings, holding classes in an online format prevents interactions among students. In addition, interactions between students and faculty members are not well established.” [S016]

A number of participants cited the online nature of the sessions and the lack of proper interaction during the sessions as factors affecting students’ motivation, fatigue, and lack of concentration.

“There are so many one-sided online meetings that bore us. I think the professors themselves get tired of this style of teaching!” [S012]

Furthermore, some students stated that online classes sometimes did not start on time. This factor lowers the students’ concentration and tranquility during the sessions.

“Often, the sessions do not start at a pre-arranged time. This disrupts our plans and causes mental tension for us!” [S023]

Most of the participants mentioned challenges related to clinical training. Participants believed that students’ clinical education was strongly influenced during the COVID-19 pandemic and distance education. In this regard, one of the faculty members suggested that:

“In the future, advances in technology may help to hold clinical classes virtually, but at present, existing technologies cannot replace student interaction with patients in the clinical environment.” [F04]

### Theme 2: opportunities

Besides the above challenges, the study’s findings indicated that online education during the COVID-19 pandemic has created several opportunities to strengthen the academic education of P&O students in Iran. Through the interviews, participants stated that online education has led to the use of new technologies, such as simulation applications, to facilitate this education style.

“The online courses have led to the use of new methods to convey educational content, such as developing applications that use artificial intelligence to simulate body movements.” [S017]

Furthermore, the development of online education-related infrastructure was another opportunity referred to by the participants. In fact, they believed that online education had provided the tools and equipment needed by universities. One of the professors stated:

“Despite the shortcomings that still exist, online classes have led to the provision of new electronic devices and tools that have increased the quality of education.” [F02]

One interesting finding was that many students believed that this teaching style was more flexible and made it easier for them to learn. Indeed, online education has prepared the environment for students and faculty members to be able to attend classes in any physical condition, an issue that many participants emphasized.

“Online education makes it possible for us to attend classes with any physical condition. It is very appropriate from this perspective.” [S03]

Regarding the student-centered learning sub-theme, the results showed that online education has led students to use more educational websites to facilitate their learning. In fact, this educational style stimulates students’ self-directed learning.

“Online education has led us to visit more educational and research sites and use their educational materials provided. Somehow, it has increased students’ self-learning” [S015]

In addition, some education platforms allow students to access educational content whenever they want. This benefit of online education was mentioned by most of the participants. They believed that this educational method makes it very easy to record and save educational content. One of the students expressed:

“Even if we are absent from the class, we still have easy access to the content of the class ....” [S08]

Time and cost savings were other benefits of online training found in this qualitative study. Participants stated that holding classes online has saved their time and reduced their costs, including travel and accommodation costs.

“We no longer have to be in so much traffic to get to university. Now we come to class very easily from home…. It saves us both time and money!” [S020]

In final, some participants stated that online education has made them more focused and confident during the sessions. In this regard, one of the students commented:

“My concentration during online meetings is very good. On the other hand, online meetings make me ask questions of the teacher with higher confidence.” [S01]

### Theme 3: recommendations

Students and faculty members suggested some recommendations to enhance the learning experiences from online education. Developing practical and user-friendly applications for clinical courses was one of the main recommendations throughout the interviews. In fact, participants believed that clinical education had been severely affected during this period, and its improvement required the development of infrastructure, including the development of applications.

“In my opinion, new systems and software should be created to facilitate clinical education in this period.” [F01]

Taking steps to improve interactions during online meetings was another suggestion. According to a faculty member, interactions among students need to be enhanced through effective teamwork during online education.

“Students need to work more closely together in online sessions and help resolve their issues through member-checking.” [F03]

In addition, many participants believed that courses could be held in hybrid form to overcome the shortcomings of online learning. This means having some face-to-face sessions to answer students’ questions and strengthen their clinical performance. One faculty member said:

“It is really hard to learn clinical topics online. I think it is better to have most clinical sessions in-person.” [S07]

In final, holding on-time sessions and raising the awareness of faculty members and students about online platforms and related digital tools were other suggestions made in this qualitative study.

“Both students and faculty members need to be more committed to starting online classes on time.” [S011]

“More training and orientation classes should be held to familiarize professors and students with how to use online platforms.” [S019]

## Discussion

With the COVID-19 pandemic, the schools of P&O in Iran rapidly moved toward online education. Although this approach had the advantage of breaking the educational interruption, it was challenging for students and professors accustomed to the in-person method. The challenges and opportunities of online education of P&O during the COVID-19 pandemic and related recommendations provided by P&O students and faculty members are discussed below.

### Challenges

#### Technical issues

One of the major challenges the study participants mentioned was internet connection issues leading to problems like connection break up, slow upload/download speed, and poor online video connections. Some participants in less developed states or rural areas faced more technical problems than those in big cities. Hardships of access to other technical requirements such as expensive laptops and smartphones, electricity shortages, and power supply problems in the country were among the other technical issues challenging distance learning, the participants of the present study explained. In addition, they believed that the platforms used for online training displayed low quality and could not meet educational needs. Restrictions to cloud computing and some educational web content like YouTube, because of filtering in Iran, have also confronted students with specific problems in storing and accessing documents and audio/video files. These contents may be used as self-learning materials, and the filtering policy deprives the students of access to them.

As a general term, online learning includes different types of education like synchronous/asynchronous online class sessions, MOOCs (Massive Open Online Courses), and interactive/passive content delivered through the internet [19]. It is usually delivered through Learning Management Systems (LMS). LMSs provide platforms for synchronous and asynchronous online education. Access to a reliable and stable internet connection with acceptable speed is one of the main ingredients of online learning infrastructure. Iran ranks 113th for median download speed according to Fastmetrics™ among 192 countries worldwide (at the time of preparing this article) [20]. Before the COVID-19 era, many educational systems had attempted to include e-learning in their educational policies and strategies to reform and transform the learning process. However, this process had not been expedited desirably in some countries. Platforms and servers that can sustain the huge workload of education for the entire system were not anticipated, especially in many developing countries. So, managing to completely transform to an online teaching-learning system with COVID-19 breakout for universities was a challenging process [21]. Similar issues regarding infrastructure and resources have been reported from online medical education in India [22]. However, in countries with more developed infrastructure, like South Korea, the students were generally satisfied with these aspects of the online learning [23]. The technical issues are reported to cause stress and anxiety to learners, especially during exams and due dates of assignments [24, 25].

#### Socioeconomic issues

Some participants cited the high cost of electronic equipment such as laptops as a significant challenge to accessing online education. Costly internet was also difficult to purchase for some participants. Holding classes at home without adequate privacy was problematic, especially for those of lower socioeconomic status.

Before the pandemic lockdown, some students with lower socioeconomic status might deal with educational demands with available facilities like public computer sites, or even there was little need to use a personal laptop, smartphone, and high-speed internet to meet daily educational needs. Online education put a significant burden on these students and their families, some of whose businesses were closed, and they had problems even with their daily income. Iran’s economy experienced concomitant stress during the pandemic due to the international sanctions. The ensuing high inflation rate resulted in a sharp rise in prices. Many students had to share their family computers. Gao et al. reported that family support had a strong correlation with e-learners’ engagement (*r* = 0.475, *p* < 0.001) [26]. Research into the issue of COVID-19 distance learning in different countries has identified socioeconomic context as an influencing factor on the students’ distance learning and learning achievements [27]. Research indicates that, in general, lower family socioeconomic characteristics may work against higher education adjustments [28]. The socioeconomic factors are related to accessibility to resources and family support, which affect the students’ e-learning experience [29]. During the pandemic, students with better access to digital resources because of their socioeconomic status of their families experienced less workload and needed less effort to achieve performance [30].

#### Environmental distractors

Since students took part in online classes without the structure of a traditional classroom, many experienced distractions when studying or attending classes. Sending and receiving text messages, opening and surfing through new tabs or social media, and other electronic devices could steal their attention. Background or outside noises like TV, music, family members’ conversations, pets, loud cars, and many other examples might interrupt the students. Sometimes other family members intrude on their privacy and make them distracted. Similarly, the professors complained of improper sound insulation in their rooms where they held the classes.

Previous studies reported that noise has negative effects on individual’s concentration and performance of complex tasks [31, 32]. Multitasking behavior and distraction can destroy the effectiveness of online classes. Online learning needs quiet space to allow the learner to engage deeply in the task [32]. Apart from quietness, other features of the place, like lighting, air quality, furniture, and temperature, are important [33].

#### Supervision and assessment

Both students and instructors had concerns regarding assessing students in online learning environments. The students believed that assessments conducted through online tests were unfair due to short response times. In addition to time management issues, unpredicted technical problems like software failure made online exams more distressing for the students. On the other hand, the professors raised concerns regarding the validity of such evaluations due to the high risk of fraud. Because weak internet connections did not support concurrent video meetings of all the students in a class, supervising the attentive presence of the students in online classes was not feasible. Tracking the activities or conversations of all the class members over class time was also a demanding task for instructors, especially for those unfamiliar with online learning interfaces.

Thorough assessments and proof of learning are generally more challenging in an online context. Most instructors had not previously practiced the specific assessment methods in an online learning environment. Applying conventional assessment methods, like summative exams, in an online learning environment encompasses more challenges. The constant availability of educational materials and contents made the students dependent on them and vulnerable to academic dishonesty. Worries about software/hardware malfunctioning and internet disconnection could increase the students’ exam anxiety. Using combined evaluation and assessment methods has been recommended by Fiesha et al. as a solution [34].

#### Workload and digital competence

Some professors explained that preparing educational materials for online classes was more time-consuming and significantly increased their workload. Being unfamiliar with devices and programs aggravated this problem.

The pandemic forced an abrupt transition from face-to-face on-site education to distance online/offline education. Neither the universities nor the students and instructors in most countries were prepared enough for this transition. This increased the students’ and instructors’ workload. Students’ workload in offline and online classes has been shown to be affected by many factors such as working hours to attend lectures, seminars, exams, project and assignments preparations, contact hours, quality of internet access, quality of contents, access to digital gadgets, educational platform, and environmental conditions [35–37]. Furthermore, adapting distance learning to the individual needs of students [2], especially the freshmen, was really a problem in this context, which may hinder the learning process. Increased workload has been suggested to contribute to the students’ stress [38]. Students’ workload has also physical aspects. Digital eye strain and fatigue due to long hours on laptops or smartphones screen [39], and awkward body postures put serious physical demands on students [40]. The sudden shift to online education has added to the stresses and workloads experienced by university faculty. Instructors had to prepare and deliver their classes from home, with all the practical and technical challenges. Many did not have any experience designing online educational content or were not familiar with the technical aspects of educational platforms [41]. The workload of their role in the family, like children’s school and learning problems, nursing a sick or elderly family member, and shared devices and places aggravate the situation [42].

#### Interaction and motivation

Several participants believed that online education greatly affected the student-student and student-professor interactions during the classes. Lack of proper interaction could lead to lower students' motivation and concentration. Such classes were boring for both students and professors.

Asynchronous courses involve little interaction between the instructor and students, which has been mentioned as a disadvantage. The perceived lack of academic social interactions affects students’ adjustment to online studying [43]. 92.1% of students who participated in Selvarej et al. survey believed that direct student-teacher interaction is essential for proper learning [44]. Some students feel lost and insecure in cyberspace because of the lack of interaction [45]. Lack of interaction may make distance learning unengaging and boring. Declining motivation in college students has been reported as one of the common effects of the COVID-19 pandemic in previous studies [46–49]. Many students struggled to stay motivated during online instructions [50, 51]. The abrupt transition to distance learning and other constraints on social activities negatively affected the students’ experience of relatedness, competence, and autonomy [46, 48, 51]. Some researchers recommended that instructors could make interventions such as defining collaborative tasks, deciding on common educational goals and preferences, and providing content consistent with goals to increase the sense of belonging in students [52]. Giving choices to students and accepting the negative emotions are among the supportive measures’ instructors can take to enhance students’ motivations [53–55].

#### Hands-on and clinical training

Most participants mentioned challenges related to students' clinical training. Available options in current online education platforms could not replace the real student-patient interaction in clinical training.

P&O is a field of practical nature. On-site education invariably consists of many hours of workshop training [11]. The undergraduate university curriculum in Iran contains more than 1800 h of hands-on and clinical training. Training in all P&O practical courses, normally organized in workshops with special equipment and instruments, totally stopped during the lockdown. This made the students experience considerable frustration. After that, the schools allowed the junior and senior undergraduate students to attend intensive programs for the practical courses on-site in small groups. This put a lot of physical and mental burden on students and the trainers and made the assessment process really hard and at risk of inadequacy. After opening the educational centers post-lockdown, students and instructors had a great workload to make up for lagging classes, making them feel discouraged or not competent enough. Online education in P&O is more complex than theoretical programs and needs collaboration among universities to share the burden of creating and preparing high-quality e-learning content for hands-on education. Innovators have taken steps in some medical fields to keep the students involved in hands-on and clinical training [56, 57]. The O&P field is a relatively small one in comparison with other majors like medicine and nursing, meanwhile with a broad spectrum of educational needs from pure theory to pure practice and skill training. Naturally, small educational budget and facilities are allocated to this subject compared to other majors with larger numbers of students. Developing specialized virtual programs, interactive environments like those available for some medical procedures, and virtual educational materials like videos and games should be custom-made and financially supported by potential sponsors. Market size and return on investment are critical determinants considered by investors. Manufacturing prostheses and orthoses and the training process are usually done in labs and workshops, and self-training is often not feasible. Although the future of P&O is inevitably toward computer-aided design and manufacturing, before and during the COVID-19 pandemic, the training was still done in conventional methods in Iran. Now that education is back to its normal form, it is recommended that universities that present O&P courses promote virtual education contents and materials. This can be fulfilled as multi-center projects to share the potential burdens. On the other hand, the transition to digitization in design and fabrication may provoke software developer companies’ positive contribution to training or help form close cooperation with universities.

#### Other parameters

Some students believed that online classes sometimes did not start on time. Supervising the online classes by universities would help better organizations. Sometimes, the lecturers experienced technical problems which may delay the schedule. A person or system in charge of managing such problems may help minimize these disorganizing events.

### Opportunities

#### Technological innovations/ infrastructure development

Participants pointed to the employment of new technologies in P&O online training during the pandemic as an opportunity. They believed that because of this, measures were taken to establish the technological infrastructure needed for online education.

Although the rapid transition from traditional to virtual education posed many challenges due to the pandemic, it brought advantages that made this threat an opportunity to modernize and improve the use of technology in education. The findings of the present study showed that although e-learning is not a new method and was used long before the COVID-19 era, only a small number of students and professors had previously experienced it. A study conducted by Carr, found that students were less satisfied with online education compared to traditional education [58]. The COVID-19 pandemic accelerated the use of e-learning and the establishment of its infrastructure, especially in developing countries such as Iran, and necessitated the use of newer technologies to hold online classes. We found that the pandemic provided an opportunity for students and their professors to become familiar with newer software and improve their skills in working with different platforms, online education, and digital content production. This finding supports the work of other studies in this area [23, 59]. A systematic review and meta-analysis showed that online education leads to increased skills in medical students [60]. In accordance with the present results, a previous study has demonstrated that virtual education provides an opportunity for teachers to acquire the skills needed to teach in a new context [61]. These experiences and increased capabilities can improve the attitude of students and teachers towards e-learning and reduce their resistance to e-learning.

#### Time and money saving/ flexible learning environment/ availability of contents

Most of this study participants stated that the independence of teaching and holding classes from a specific place and environment made them feel better and enabled them to attend classes from home without needing transportation. This has been the most advantageous for them and has saved them a lot of time and money, in addition to slowing down the transmission of the virus. Ease of access to educational materials is another benefit of e-learning. Documenting and recording class materials allows students to access educational content whenever they want. Previous research has also found that students prefer e-learning because of its flexibility in time, place and access to its content [56]. This helps students to be more focused when studying educational content.

#### Student-centered learning/ more students’ self-confidence

Many participants acknowledged that the COVID-19 pandemic provided the opportunity to increase their self-learning skills. Some students stated that online learning increased their self-confidence and reduced their fear of involving in class activities. In our study, graduate students were more satisfied with online education. This may be related to the greater motivation of these students to improve their self-study skills. It is encouraging to compare this finding with that found by Stacey and Gebric [62], who found that students’ motivation can increase their self-study skills. Surveys such as that conducted by Liaw et al. have shown that increasing self-learning and e-learning skills improves problem-solving skills and critical thinking in individuals [63]. Another advantage of e-learning was the increase in self-confidence of some participants. This allowed them to be more engaged in class activities with less fear of making mistakes. A study on 431 undergraduate and graduate students in 30 different disciplines showed that holding classes online during COVID-19 led to less pressure and stress on students during exams [64].

### Recommendations

#### Hybrid courses/ technical infrastructure/ team dynamics

Students participating in the present study noted that they did not have sufficient self-confidence and skills to make orthoses and prostheses without attending the workshop. They also believed that this condition lowers their self-confidence in dealing with patients and fitting orthoses or prostheses for them. They believed that for virtual technologies to be considered a long-term and realistic solution, significant infrastructure improvements must be made, especially for clinical purposes, to meet individuals’ clinical learning needs. According to students, using the hybrid method, namely holding both face-to-face and online classes can be helpful. In addition, teachers believed that in the future, different methods, such as team-based or group-based learning, should be used to increase student participation and interaction.

Many undergraduate and postgraduate P&O courses should be held practically. This requires students’ attendance in the workshop to learn to work with tools and the stages of making different orthoses and prostheses. Therefore, relying on e-learning alone is not enough to meet the learning needs of students, and similar models of theory lessons cannot be used in this area. A survey of 1,289 students and faculty members in Saudi Arabia indicated that despite the challenges of online education, including inexperience in online education, time management, stress caused by COVID-19, and technophobia, 62% preferred the combination of online and face-to-face training programs [65]. Other researchers have also shown that the use of e-learning and simulated learning environments are among the distance learning methods that have positive results in improving the clinical competency of rehabilitation students [66].

### Limitations

Despite all efforts, participants were not from all P&O faculties offering this program in Iran. Most participants were students of educational institutes in Tehran (the capital of Iran). P&O program in other faculties has been founded relatively recently, and their students may have experienced different issues. The results of this study could also be improved by reviewing the relevant literature.

## Conclusion

Online education of P&O during the era of the COVID-19 pandemic was accompanied by a series of challenges and opportunities which are generally seen in this type of education. However, the great proportion of hands-on education in this field is a particular challenge in the context that needs to be dealt with. Considering hybrid (mixed online and on-site) courses was recommended to improve the quality of learning. Designing and implementing virtual practical training programs with the contribution of all the departments involved in P&O academic education is recommended.

## Data Availability

The datasets used and/or analysed during the current study are available from the corresponding author on reasonable request.
